# The Sibylline Relationship Between Human Papillomavirus and Endometrial Cancer: Scarcity of Strong Evidence Linking Both Conditions

**DOI:** 10.3390/v17050607

**Published:** 2025-04-24

**Authors:** Khadija Bichri, Adil El Ghanmi, Fadila Kouhen, Salsabil Hamdi, Karima Fichtali, Fadoua El Mansouri, Jalila El Bakkouri, Bouchra Ghazi

**Affiliations:** 1 Immunopathology-Immunomonitoring-Immunotherapy Laboratory (3Is), Faculty of Medicine, Mohammed VI University of Sciences and Health (UM6SS), Casablanca 82403, Morocco; kbichri@um6ss.ma (K.B.); aelghanmi@um6ss.ma (A.E.G.); karima.fichtali@yahoo.fr (K.F.); elmansouri99@yahoo.fr (F.E.M.); jelbakkouri@um6ss.ma (J.E.B.); 2 Department of Gynecology and Obstetrics, Mohammed VI International University Hospital, Bouskoura 27182, Morocco; 3 Laboratory of Neurosciences and Oncogenetics, Neurooncology and Oncogenetic Team, Faculty of Medicine, Mohammed VI University of Sciences and Health (UM6SS), Casablanca 82403, Morocco; fkouhen@um6ss.ma; 4 Department of Radiotherapy, International University Hospital Cheikh Khalifa, Casablanca 82403, Morocco; 5 Virology and Public Health Laboratory, Institut Pasteur du Maroc, Casablanca 20360, Morocco; salsabil.hamdi@pasteur.ma; 6 Clinical Immunology, Inflammation and Allergy Laboratory (LICIA), Faculty of Medicine and Pharmacy, Hassan II University, Casablanca 20250, Morocco

**Keywords:** endometrial cancer, human papillomavirus, carcinogenesis, viral replication

## Abstract

Endometrial cancer (EC) is the fourth-most frequent cancer among the female population and a leading cause of death. Multiple factors are susceptible to causing tumorigenesis, including obesity, lack of physical activity, diabetes mellitus, high concentration of estrogen during menopause, unopposed exposure to estrogen, duration of menses, nulliparity and infertility. Human papillomavirus (HPV) is a double-stranded DNA virus, with certain genotypes exclusively human. HPV plays a major role in some cancers (cervical cancer, head and neck cancer, lung cancer, and anogenital cancers). Given the intricate correlation between HPV and cervical cancer, the scientific community conjectured that HPV may be implicated in the carcinogenesis of the endometrium. In this review, we will direct our interest towards previous studies that focused on the expression of HPV on EC samples and cover how both conditions might connect to each other.

## 1. Introduction

Endometrial cancer (EC) is a disease occurring only in the female population, restricted to the uterus corpus. This adenocarcinoma emerges from intrauterine epithelial cells. The tumor microenvironment overlaps multiple types of cells, such as stromal cells, endothelial cells, and immune cells [1]. The existing interactions in the tumor microenvironment promote the proliferation of cancer cells, by the production of energy-rich catabolic metabolites [2].

In the United States, EC is considered the fourth-most common cancer, with 66,200 estimated new cases and 13,030 deaths in 2023 [3]. According to the GLOBOCAN estimation, EC was diagnosed in 417,367 women in 2020 [4]. The disease’s incidence was especially observed in developed areas, with China, the United States, and Russia being the top three of the most touched countries as mentioned by the World Cancer Research Fund International [5]. It is predicted that an increase of 40–50% in endometrial cancer incidence will be assessed over the decades [6].

Ethnicity is also involved in the induction of endometrial cancer, as the incidence rate of the disease was more prominent in white women. On the other hand, the mortality rate was higher in black women [7,8].

Multiple risk factors are culprits for the manifestation of endometrial cancer, including obesity, diabetes mellitus, unopposed exposure to estrogens, duration of menses (early age of menarche or late age of menopause), nulliparity, high postmenopausal concentration of estrogens, and lack of physical activity [9]. Some studies suggest that night sleep deprivation could also lead to the development of endometrial cancer. Still, no significant association was found between the disease and the deregulation of the circadian cycle [10].

The Human papillomavirus, a double-stranded DNA virus, is the cause of a plethora of cancers, including cervical cancer, head and neck cancer, lung cancer, and anogenital cancers [11]. Specifical genotypes, categorized as high-risk HPV were mostly associated with tumorigenesis [12], notably HPV-16, 18, 31, 33, 35, 39, 45, 51, 52, 56, 58, and 59. Given the proximity of the endometrium to the cervix, numerous investigations were led to find a possible association between human papillomavirus and endometrial carcinoma. The results of these studies are controversial due to certain factors, such as the diversity in the methods used by scientists.

In the present review, we will enumerate the different factors that emphasize the carcinogenesis of the endometrium, and cite the classification of EC. Subsequently, we will recapitulate the HPV’s replication mechanism and scrutinize anterior research inspecting its eventual relationship with EC.

## 2. Risk Factors of Endometrial Cancer

**Figure 1:**
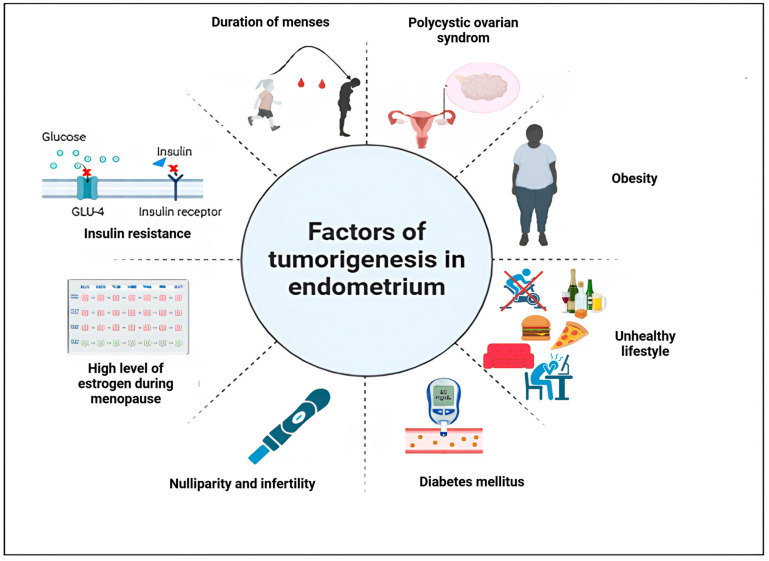
Risk factors of endometrial cancer. Figure created using Biorender. https://app.biorender.com/illustrations/67a682280d0da18dcce77763 (accessed on 23 July 2024).

Multiple risk factors have been held responsible for carcinogenesis in the endometrium (Figure 1). According to the World Health Organization (WHO), obesity is defined as an excess deposit of fat, leading to type 2 diabetes, heart disease, affection of reproduction, and bone health. Moreover, it is incriminated in increasing the risk of cancer [13]. Body mass index (BMI), calculated with the formula (BMI = weight (kg)/height^2^ (cm)), is used for diagnosing obesity and determining its grade. Someone is qualified as obese if their BMI is >30 kg/m^2^, corresponding to the WHO classification.

With the meta-analysis realized by Zhang et al., [14], it is clear that obesity is positively correlated to endometrial cancer. The mechanisms by which obesity induces endometrial cancer are described as follows: the conversion of androstenedione, an androgen, to estrone, with a decreased serum level of sex-hormone binding globulin (SHBG) will create an environment enriched in estrogen and thus stimulate the development of endometrial cancer cells [15]. The transformation of androgens to estrogens in postmenopausal women in peripheral fat stores is also responsible for endometrial cancer, as it stimulates the proliferation of endometrial cells by blocking apoptosis and promoting angiogenesis [16]. Additionally, insulin resistance increases the risk of developing endometrial cancer. Insulin stimulates ovarian androgen synthesis and regulates hepatic synthesis and plasma levels of SHBG, which will affect serum estradiol levels [15]. Furthermore, certain cytokines produced by adipose cells are related to insulin resistance, as adiponectin decreases serum glucose concentration and leptin inhibits signaling through insulin receptors, reducing insulin response [17]. All these processes are prone to causing endometrial cancer [18]. Diabetes is characterized by metabolic disorders, such as insulin resistance, hyperglycemia, and hyperinsulinemia [19]. According to the Global Burden of Diseases, Injuries, and Risk Factors Study, this chronic disease was a leading cause of mortality, with 460 million deaths worldwide [20]. Type 2 diabetes mellitus (T2DM) is associated with an increased risk of EC, in the contrary of non-diabetic cohort, as shown by Zhang et al., [21].

Hyperglycemia, a common comorbidity of obesity, is incriminated in the growth of the tumor, as it provides energy to different metabolic pathways [22]. A myriad of abnormalities have been assessed in EC, such as the loss of PTEN, leading to the activation of the PI3K/AKT/mTOR pathway, therefore initiating EC in mice [23]. Other aberrations, namely mutation in KRAS, overexpression of EGFR, stimulation of the Wnt/β-catenin pathway, and mutations in TP53 [24] enhance the glucose metabolism, through the regulation of glucose transporters (GLUTs 1, 3, 6 and 8) and the alteration of enzymes regulating glycolysis [25]. Certain of these proteins, involved in insulin signaling, join the PI3K/AKT/mTOR pathway, which plays a major role in glucose metabolism, anabolic cell growth, proliferation, survival, metastasis, and drug resistance [22]. With all these mechanisms involved in the growth of EC, it is clear that hyperglycemia is associated witha higher risk of EC.

Lifestyle and nutrition regimens are involved in the etiology of endometrial cancer. Bad eating habits and a diet poor in nutrients are factors for the apparition of the malignancy [26]. Pieces of evidence indicate that physical activity is a way to reduce EC risk by decreasing serum levels of estradiol and SHBG [27]. Therefore, physical activity is a good strategy for preventing EC and obesity.

It is commonly known that EC is a hormonally responsive tumor. At the beginning of the 1960s, the prescription of estrogen therapy helped women deal with menopausal symptoms and prevent osteoporosis and heart disease [28]. However, the increase in sales of estrogens associated with the rising incidence of EC in the 1970s led to a possible relationship between the two phenomena [29]. This causality effect was the origin of the unopposed estrogen hypothesis, which stipulates that the exposure of endometrium to estrogen only, without progesterone, can promote endometrial cell growth and, thus malignant transformation [30]. Key and Pike [30] showed that exposure to estrogen without progesterone leads to a dysregulation of the mitogenic effects of estrogen, with uncontrolled endometrial proliferation during the follicular phase. This may lead to alterations in proto-oncogenes and tumor suppressor genes [31], and ultimately to EC.

Another culprit for the unbalance between estrogen and progesterone is polycystic ovarian syndrome (PCOS), characterized by androgen excess, menstrual abnormalities, and ovarian cysts [32]. Two mechanisms underlying PCOS are responsible for EC. On one hand, the dysregulated menstrual cycle will increase the duration of estrogen exposure and conduct endometrial carcinogenesis [33]. On the other hand, PCOS is related to insulin resistance and hyperinsulinemia [34]. Furthermore, the conversion of androgens into estrone, due to hyperandrogenism, increases the rate of circulating estrogen [35]. All these pieces of evidence show that high estrogen levels, unopposed by sufficient progesterone, are responsible for EC.

According to Petterson et al. [36], the cause of the disease could be attributed to the number of ovulatory cycles due to the early age of menarche. Parallel to this finding, Felix et al., [37] showed that older menarcheal age (≥15 years old) is inversely associated with the occurrence of EC. Moreover, longer exposure to estrogen due to late menopause increases the risk of EC. As demonstrated by Wu et al., [38], women whose menopausal age is above 46.5 years old are more likely subject to develop endometrial cancer.

As shown by Chen et al., high levels of progesterone, concomitant to estrogen in parous women during pregnancy, lower the risk of EC [39]. Additionally, the addition of a child after the second birth decreases the risk of developing the disease by 10%, a phenomenon being explained by the hormonal imbalance created by pregnancy, thus reducing the mitotic effect of estrogen [40]. Furthermore, nulliparity and infertility are risk factors for the development of the malignancy [41], due to long exposure to estrogen [42].

The totality of these factors is involved in the development of endometrial cancer. However, some of these characteristics could be mitigated, like obesity and diabetes, by choosing a healthy diet and frequent physical activity.

## 3. Classification of Endometrial Cancer

The first classification of EC was initially proposed by Bokhman. The malignancy was classified into two groups: type I, associated with unopposed estrogen stimulation and linked to a favorable prognosis, whereas type II was not estrogen-driven, and had a worse prognosis [43]. Type I englobes low-grade (grade I and II) endometrioid adenocarcinoma in contrast to type II, which overlaps grade III endometrioid adenocarcinoma, serous clear cell, undifferentiated carcinoma, and carcinosarcoma.

With the recent update in the World Health Organization’s classification of female genital tract tumors (2020), endometrial cancer has been classified into eight different histological types (Table 1) [44].

In 2013, The Cancer Genome Atlas (TCGA) categorized EC into four subgroups. This classification was based on the copy number alterations and the mutational burden [46]. The study, which focused on 373 cases of serous and endometrioid endometrial cancer, helped determine the prognosis of each group (Table 2) [47].

Due to the difficulty of applying this classification in clinical practice, a new simplified system has emerged to determine the prognosis of patients: the proactive molecular risk classifier in endometrial cancer (ProMisE) [49]. The ProMisE classification is based on the use of molecular detection of the POLE exonuclease domain mutation (POLE mut) and immunohistochemical markers: wild-type p53 (p53 wt), p53 abnormality (p53 abn), and mismatch repair proteins (MMR). These factors stratified endometrial cancer into four subgroups: POLE mut, MMRd, p53abn, and NSMP (Table 3).

## 4. FIGO Classification

The updates brought in the classification of endometrial cancer by the International Federation of Gynecology and Obstetrics (FIGO) in 2023 consider new criteria opposite to the classification of 2009. The addition of lymphovascular space invasion, lymph node status, and molecular classification defined the new subtyping of FIGO. Furthermore, these criteria helped guide toward more appropriate therapeutic approaches. Histologically, the FIGO splits EC into endometrial endometrioid carcinomas (EECs) and non-endometrioid carcinomas (Table 4) (NEECs).

The lymphovascular space invasion (LVSI), one of the new characteristics of the FIGO 2023 classification, is a determining factor of stage II EC [61]. In clinical practice, LVSI has a major role in guiding adjuvant therapy decisions [62]. It is thus important to differentiate between LVSI and its mimickers, such as microcystic elongated and fragmented pattern of myometrial invasion and retraction artifacts [63]. The new classification insists on the importance of defining the extent of LVSI, as focal or the absence of LVSI is a synonym for good prognosis [64], whereas substantial LVSI is linked to poor prognosis [61]. According to the WHO, substantial LVSI corresponds to five or more instances of LVSI [44,65].

A new, noninvasive way of determining the sentinel lymph node (SLN) has been adopted: the SLN mapping, as it stages endometrial cancer and detects low volume metastasis (LVM). The 2023 FIGO staging separates lymph node status into two categories: micrometastasis, and isolated tumor cells (encompassed in LVM) and macrometastasis (Table 5) [60].

The addition of the molecular subtyping accentuates the precision of the prognosis. Determining which molecular subtype (POLE mut, MMRd, p53abn, and NSMP) is a key factor for choosing the appropriate adjuvant therapy [60].The detection of POLE mut or p53abn subtypes in the early stages of endometrial cancer is now a main criterion for 2023 staging. Regardless of the cervical involvement, LVSI status, or histological type, an ancient POLE mut subtype is now categorized as a stage IAmPOLEmut [60]. The same applies for a p53 abn subtype, as the new classification considers it a stage IIC2mp53abn [60].

## 5. Human Papillomavirus (HPV)

As a member of the Papillomaviridae family, the ability of the Human Papillomavirus (HPV) to hide from the immune system of the host makes it a tough opponent to eliminate. The WHO considers infection by this virus a global threat to public health, as it could evolve into some types of cancer [67]. In 2019, the estimated rate of cancer caused by HPV among women was 62,000 new cases, whereas in men, the cases were 70,000 [68].

**Figure 2:**
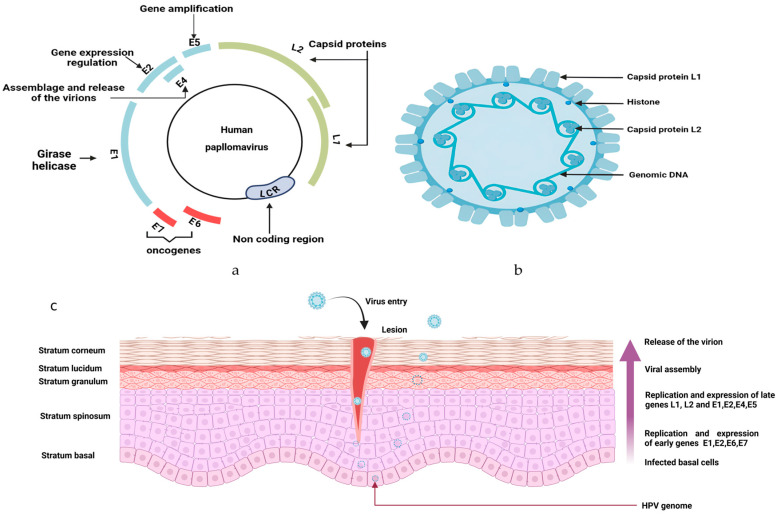
Structure of human papillomavirus: (**a**) The genome of HPV is circular, with 6 early regions (E1, E2, E3, E4, E5 and E6) and 2 late regions (L1 and L2). In high-risk HPV, E6 and E7 are oncogenic proteins. While the early regions are specific to the regulation functions, the late regions are for the virus transmission. The long coding region (LCR) is a noncoding region. (**b**) Internal structure of HPV. (**c**) HPV infection and life cycle in the stratified epithelium: Through an abrasion occurring in the skin, HPV infects the cells in the basal stratum. The virion is subject to proliferation via DNA replication and has an epigenomic form in the nucleus of the cell. Early genes E1, E2, E6, and E7 are expressed, leading to productive genome amplification. Migration of infected basal upwards to induce epithelial differentiation. The assemblage of the virion happens in the upper layer of the epithelium, due to the expression of late genes L1 and L2. Figure created using Biorender. https://app.biorender.com/illustrations/67a9af17735e9ea7738a45a6 (accessed on 21 September 2024).

HPV is a double-stranded DNA virus, non-enveloped. More than 170 different genotypes have been discovered and split into two categories: low-risk HPV (LR-HPV) and high-risk HPV (HR-HPV) [69]. LR-HPVs are mostly responsible for anogenital or cutaneous warts and can scarcely lead to cancer [70]. However, HR-HPVs are more likely apt to develop malignancies, such as oropharyngeal cancers, and anogenital cancers (anal, cervical, vaginal, vulvar, and penile tumors) [71]. HPV is an isohedral virus, with only one strand of its DNA made of eight opening reading frames: six early regions (E1, E2, E4, E5, E6, and E7) and two late regions (L1 and L2) (Figure 2) [72].

The transmission of HPV is mainly through sexual or skin-to-skin contact, and also through perinatal contact [73]. The tropism of the virus is for epithelial cells, as human HPV targets specifically differentiated squamous epithelium [74]. To permit proper entry to the nucleus, the virus requires active cell division of basal keratinocytes [75]. After its translocation to the nucleus, the virus triggers its replication and transcription. For HR-HPV, the binding of the viral DNA to the host cell nucleus disrupts cell growth and differentiation by dysregulating oncoproteins (E6 and E7). The mutual inactivation of p53 and pRB (retinoblastoma protein) is responsible for anarchic cell proliferation and inhibition of the function of cell cycle checkpoints [74,76]. The degradation of p53 is done through the formation of the complex p53-E6-E6AP, leading to the loss of the function of p53. In normal conditions, p53 stops the G1 phase of cells during mitosis and initiates apoptosis to allow host DNA repair [11]. Once E7 binds to pRB, it induces its degradation, altering its function of cell-cycle control [77]. This whole process is incriminated in carcinogenesis and determined HPV as a culprit in different types of malignancies [78].

## 6. Human Papillomavirus in Cancer

Human papillomavirus has been determined as a culprit for carcinogenesis in head and neck squamous cell carcinomas (HNSCC), anogenital cancers, cervical cancer, and lung cancer. In HNSCC, it has been demonstrated that the virus modulates innate and adaptative immune responses. The biology of this parasite allows its integration into the host cell genome or to stay in an episomal form [79].

The entry of the virus in the cell is facilitated by the L1 and L2 regions, in addition to the interaction with cellular components, such as heparan sulfate proteoglycans, which play the role of capture molecules [80]. Furthermore, syndecan-1, a heparan sulfate proteoglycan involved in the communication between extracellular matrix and cells [81], is to date, the only known binding receptor of HPV [82]. This transmembrane proteoglycan is incriminated in malignancies, such as myeloma, medulloblastoma, endometrial cancer, and breast cancer by helping their proliferation, modulating the cell cycle, and enhancing angiogenesis [83,84,85,86]. Szatmári et al. demonstrated that syndecan-1 translocated to the nucleus presented an anti-proliferative function, while it dysregulated the TGF-β-signaling pathway and the cell cycle. Three target genes were retrieved in the study; EGR-1, NEK11, and DOCK8, as all of them are implicated in the regulation of the cell cycle [87]. Moreover, syndecan-1 is linked to NFϏβ, as its knockdown decreases the cellular proportion of the latter [88].

First, the virus penetrates the basal lamina of the stratified epithelium through microlesions [89]. The virus attaches itself by binding its L1 capsid protein to the surface of basal keratinocytes [90], marking this step as the inducer of modifications in the capsid structure and allowing the binding of L2 protein capsid to secondary receptors [91]. The virus enters the cell via endocytosis, followed by the capsid’s dismantling and the viral genome’s orientation, along with L1 and L2, to the trans-Golgi network [92]. Once the virus breaks into the nucleus of the host’s cell, its genome binds to the host’s chromosomes, permitting viral amplification [75]. However, there is the maintenance of minimal virus protein expression due to the viral protein E2 [93]. The oncoproteins E5, E6, and E7 are held responsible for the late differentiation and cell cycle exit, as they promote viral amplification. E5 can influence the immune response following the infection by causing the underexpression of major histocompatibility complex (MHC) I, MHC II, and CD1d at the surface of T cells [94,95,96]. Moreover, E5 suppresses STAT1, causing a defect in INF signaling and repressing the expression of INFκ, which has a role in antitumoral immunity [97]. E5 inhibits apoptosis to prevent the death of HPV-infected cells by inducing the degradation of the pro-apoptotic Bcl-2 family member BAX [98], decreasing the expression of the Fas receptor and impeding the recruitment of Fas-associated protein with the death domain (FADD) [99].

E6 helps accelerate cancer development, as it disturbs host signaling pathways. Indeed, when E6 binds to its receptor E6-associated protein (E6-AP), the complex suppresses the proteasome NHERF1, which negatively regulates the Wnt pathway, thus allowing the accumulation of β-catenin and therefore the transcription of proliferative genes [100]. The Notch signaling pathway is involved in cell division, differentiation, and survival [101]. The binding of Notch receptors to their ligands will lead to cleavage in the receptor. Subsequently, the release of the Notch intracellular domain (NCID) permits its translocation to the nucleus to activate the transcription of target genes [102]. The Notch pathway has presented both oncogenic and antitumoral properties in cancers, with its dysregulation being a factor for angiogenesis and epithelial-mesenchymal transition [103]. Additionally, through the degradation of the TP53 tumor suppressor, E6 downregulates Notch1 receptor in cervical cancer, which inhibits the growth of HPV-positive cervical carcinoma cells [104]. As shown by Rangarajan et al., the activation of Notch 1 works along with oncogenes of HPV to induce immortalization of epithelial cells, leading to resistance to apoptosis and anoikis through the activation of a Ras effector (PKB/Akt) in cervical cancer [105]. However, another study led on genetically engineered mouse models (GEMs), showed that the inactivation of Notch pathway in head and neck squamous cell carcinomas, with active HPV16 E6 and E7 oncoproteins, led to tumorigenesis with an accumulation of β-catenin in the nucleus [106]. The maintenance of cell homeostasis is due to the PI3K/Akt pathway, as it controls cell growth and survival [107]. Nonetheless, the modulation of the PI3K/Akt/mTor pathway by HPV oncogenes (E6 and E7) is another event related to carcinogenesis, as the overexpression of PI3K in cervical cancer is associated with the proliferation of cells and their resistance to apoptosis [108]. Furthermore, the secretion of IL-17 due to HPV infection in lung cancer stimulated the expression of Mcl-1, an antiapoptotic gene, through the PI3K pathway to promote tumorigenesis [109].

Viruses and hosts can live together in symbiosis. The healthy human virome encompasses Papillomaviruses. Indeed, Beta and Gamma HPV types can be found in healthy skin without causing any apparent disease or can evolve into small infections that can be eliminated by the organism [110]. However, the Alpha papillomavirus is the type incriminated in the apparition of malignancies as it infects mucosal tissues, causing cervical, anogenital, and head and neck cancers [111]. High risk HPV is able to induce squamous intraepithelial lesions that could develop into invasive squamous cell carcinoma through the expression of its oncogenes E6 and E7, and the amalgamation of the viral genome into the host genome [112,113,114]. HPV is highly prevalent in reduced-immunity populations, namely HIV patients and in kidney transplant recipients [115,116]. HPV is fit to escape the physical barrier of the host by metamorphosing its conformation via cell surface cyclophilin B [117], flee the innate and humoral immunity by reducing the production of antigens [118] and by using E6 and E7 for the degradation of p53 and Rb, which are associated with immunity [119].

**Figure 3:**
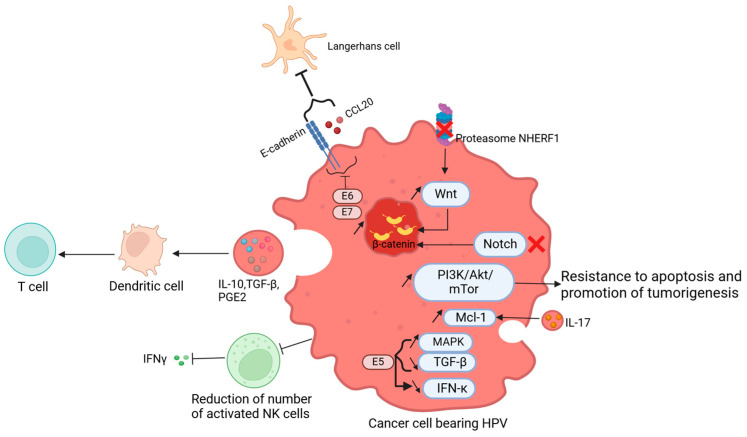
Tumor microenvironment modulated by HPV: HPV influences the tumor microenvironment, to maintain the survival of infected cells. The degradation of the proteasome NHERF1 leads to an upregulation of the Wnt pathway and the accumulation of β- catenin. This accumulation was also observed after the inactivation of the Notch pathway, therefore, there is a promotion of the tumor. Moreover, the production of IL-17 stimulates Mcl-1, through the PI3K/Akt/mTor pathway, causing resistance to apoptosis and tumor development. The number of activated NK cells is decreased, causing the inhibition of INF-γ. The increased levels of IL-10, TGF-β, and PGE2 are related to the immature state of dendritic cells and subsequently the lack of activation of T cells. The HPV E5 protein leads to the overexpression of MAPK, with a decrease inTGF-β, and therefore a decrease in IFN-κ. The oncoproteins E6 and E7 reduce the E-cadherin and CCL20, diminishing the number of Langerhans cells. Figure created using Biorender. https://app.biorender.com/illustrations/669e61f27e009608e0293e27 (accessed on 22 July 2024).

In cancer, HPV can influence the tumor microenvironment in a paracrine manner, to avoid the senescence of HPV-bearing cells [120] (Figure 3). Keratinocytes, which are sentinel cells, possess pathogen recognition receptors (PRRs), which are essential for a cascade of immune reactions against a pathogen [121]. However, in the case of HPV infection, the virus acts to inhibit this cascade of reactions, allowing its survival through the blockade of secretion of cytokines [122]. The decreased expression of TGF-β, balanced by the overexpression of MAPK, due to the HPV16 E5 oncoprotein leads to a diminished expression of IFN-κ [97]. However, in oropharyngeal squamous cell carcinoma, levels of TGF-β, IL-10, TNF-α, and VEGF were increased compared to controls, showing the importance of these cytokines in promoting the tumor [123]. Additionally, studies have shown a higher production of IL-10 by HPV16 E2 protein, helping the virus to subsist in infected cells [124]. At the immune cell level, HPV reshaped the immune response to form a suitable microenvironment for its survival. The Langerhans cells, which represent the antigen-presenting cells of the epidermis, are not efficient in cases of HPV infection due to the underexpression of CCL20 and E-cadherin, a molecule allowing the adhesion of the Langherans cells on keratinocytes [125]. Moreover, dendritic cells, another population of antigen-presenting cells, are downregulated due to the secretion of immunosuppressive cytokines by keratinocytes, namely: IL-10, TGF-β, and PGE2 [126,127]. Therefore, the lack of antigen presentation leads to the non-activation of T cells.

On the other hand, HPV can recruit immunosuppressive cells in lesions in cervical or head and neck squamous carcinomas, such as tumor-infiltrating lymphocytes (TAMs) [128,129], and myeloid-derived suppressive cells (MDSCs). The latter subset of cells produces arginase 1, reactive oxygen species, indoleamine-2,3-dioxygenase, and immunosuppressive substances [130]. Natural killer cells (NK) are also downregulated by the persistent infection by the virus, leading to the inhibition of IL-18-dependent IFN-γ production [131,132]. Thus, NK cells are inapt to eliminate infected cells. The ability of T cells to eradicate pathogens is through the recognition of antigen peptides presented by antigen-presenting cells, and the differentiation into CD8+ cytotoxic cells. Nonetheless, in cancerous lesions, cytotoxic T lymphocytes have no antitumor activity towards malignant cells. This phenomenon was explained by the decreased expression of MHC-I on keratinocytes’ surfaces or the lack of maturation of Langerhans cells (Figure 3) [133,134,135].

## 7. Human Papillomavirus and Endometrial Cancer

The evidence of carcinogenesis due to HPV was assessed in multiple studies during the last century. Two types of high-risk HPV were particularly raising suspicion among the scientific community. HPV-16 and 18 were the most implicated in tumorogenesis, as Zur Hausen highlighted in 1996 [136]. These two HR-HPVs were associated with cervical [137] and anogenital cancers [138] as well as esophageal squamous cell carcinoma [139], lung cancer [140,141], and head and neck cancer [142].

HPV-associated cervical cancer is one of the most spread diseases among the female population, occupying the fourth position among leading causes of cancer-related death [4]. The virus is also linked to other tumors occurring in the female reproductive tract, such as vulvar and vaginal cancers [143]. As the cervix is constituted by stromal cells and epithelial cells, the response to pathogens is controlled by sex steroid hormones and oscillates between the edification of an epithelium barricade [144] and the secretion of mucus [145] and immune cells. The cervical epithelium blocks the pathogen entry to the upper female reproductive tract by tightening the junction between adjacent cells [146], producing a mucus composed of various elements (mucin, lipids, immunoglobulins, lipids, fatty acids, enzyme inhibitors, enzymes, trace metals, proteins) [147], and secreting chemokines, cytokines, and antimicrobial peptides [148,149,150]. On the other hand, the endometrium, which is formed by epithelial, stromal, immune, and vascular cells [151], is also protected by antimicrobial peptides [152] and Toll-like receptors acting as pattern recognition receptors for antigens [153]. The suggestion that endometrium is a “sterile” environment due to the protection imposed by the cervix was refuted [154,155]. Given the role of HPV in the apparition of malignancy in the cervix, the scientific community decided to verify whether the virus could also lead to carcinogenesis in the endometrium.

As HPV can infect the cervix, researchers looked into the possibility that the virus could infect the endometrium and be etiological in endometrial cancer [156]. Another factor hinted at the eventuality of HPV as a culprit for type 1 endometrial cancer: the infection by the virus increases rates of estrogens [157].

The studies focusing on the relationship between HPV and endometrial cancer are controversial, as some researchers found no evidence of malignancy being influenced by viral charge. On the other hand, others detected HPV in samples of endometrial cancer. In the late 80s, Bergeron et al. looked for HPV in 28 endometrial samples by using Southern blot hybridization [158]. However, no HPV DNA was identified among the endometrial tissues. Another study, in the Moroccan population, aimed to highlight the possible involvement of HPV in endometrial cancer by using Polymerase chain reaction (PCR) in biopsies from hysterectomy and myomectomy, to detect several genotypes of the infectious agent: HPV 6, 11, 16, 18, 31, and 33. No association was found between the viral microorganism and endometrial cancer [159]. Several other researchers investigated the possible link between HPV and endometrial carcinoma, nonetheless, the results were the same as mentioned earlier [160,161,162].

Mahmoud and Rifat [163] used real-time Polymerase chain reaction (RT-PCR) to detect HR-HPV in 90 patients (30 with endometrial hyperplasia, 30 with endometrial carcinoma, and 30 cases of hysterectomy). Despite the presence of HR-HPV in 23 patients (mainly in endometrial hyperplasia), the pathogenesis of EC could not be attributed to HPV.

According to Grabarek et al., infection with HPV 16 or 18 was an increasing risk factor for developing endometrial and ovarian cancer, however not all the results were significant [164]. These findings relate to the low rate of HPV among patients with endometrial malignancy [165,166,167], showing a non-significant correlation between viral infection by HPV and endometrial carcinoma.

On the other side, Fujita et al. and Wu et al. insist on further analysis of the role of HPV in endometrial carcinogenesis. The study of Wu et al. [168] shows an increased risk of type 1 endometrial cancer among the cohort that is positive for HPV. On the same note, Fujita et al. [169] found HPV DNA in 6 of 47 endometrioid carcinomas from Japan and 2 of 38 endometrioid carcinomas from the United States. These results led the team to think that HPV 16 may have a role in endometrioid adenocarcinomas.

As emphasized by Giantromanolaki et al., HPV is not an initiator of endometrial adenocarcinoma, even with the detection of the virus in the samples. The endometrium may not be a host for the multiplication of HPV, as there are no epithelial changes proper to the viral infection. These conclusions show that HPV originating from the lower female genital tract is temporary in the endometrium and has no role in its pathogenesis [165].

The incidence rate of cervical cancer caused by human papillomavirus has decreased in a significative manner, despite the increased carcinogenesis targeting the oropharyngeal and anal/rectal zones due to the virus, in the male population [170]. Traditional methods of detection of HPV encompass real-time or reverse transcriptase polymerase chain reaction, Southern blot, staining of the p16, and in situ hybridization, while new methods are also emerging to reduce the cost or enhance the specificity, such as the nucleic-acid-amplification-free electrochemical biosensor based on the CRISPR/Cas9 technology [171,172]. The search for an agent responsible for the induction of a malignancy in the endometrium has been active in the last few decades, as several studies tried to uncover the possible link between viruses and EC. Indeed, hepatitis B, hepatitis E, measles virus, and human cytomegalovirus were found in EC samples, which raised the question of whether any of these viral agents are linked to the tumorigenesis [164,173,174,175]. Furthermore, as microbiota has been studied in various solid malignancies, studies were engaged to investigate thoroughly the correlation between microbiome and EC. Indeed, in endometrial malignant tissues, the abundance of *Nitrilirupter* and Blautia bacteria is linked to a poor EC prognosis [176]. Another article highlighted the diminished cervicovaginal and rectal bacterial mass along with the decreased prevalence of *Lactobacillus* species and the increase in *Porphyromonas*, *Prevotella*, *Peptoniphilus*, and *Anaerococcus* [177]. Thus, it would be of great interest to further investigate the possible agents suspected in tumorigenesis of the endometrium to confirm a possible correlation between them.

No real connection was found between HPV and endometrial cancer despite the presence of the virus DNA in samples of the carcinoma. Thus, the qualification of HPV as a “passenger” in the endometrium by Giantromanolaki et al. is a valid hypothesis.

## 8. Conclusions

In the end, the studies investigating the role of HPV in the carcinogenesis of the endometrium are controversial. However, shreds of evidence showing no real association between the virus and the pathogenesis are more numerous. The conflicted findings in the literature may be explained by the different methods of detection used, the tissue preparation techniques, or the different populations studied. Thus, it is important to detect HPV antigen in endometrial cancer samples and use more precise techniques to detect the DNA of the virus. Moreover, the actual evolution known by artificial intelligence could be key as a non-invasive method of detecting the virus. The development of recent deep learning and machine learning models for the detection of HPV in head and neck cancers has been promising. Therefore, it would be interesting to introduce new machine learning models for the detection of HPV in endometrial cancer and know for sure if the infection by this virus is associated with carcinogenesis in the endometrium. Furthermore, the induction of carcinogenesis in the endometrium could be caused by other agents—notably by dysbiosis in the uterine tract, causing an unbalance in the acidity of the milieu, and the production of pro-inflammatory cytokines—in addition to the dysregulation of signaling pathways such as PI3K/AKT/mTOR, WNT/β-catenin, and MAPK/ERK pathways. The carcinogenesis in the endometrium may also be induced by other potential viruses, including measles virus, hepatitis B, and hepatitis E, although these hypotheses remain to be studied further.

## Figures and Tables

**Table 1:** Histological classification of EC according to the World Health Organization (5th edition of the WHO classification of tumors: female genital tumors) [45].

Aggressiveness	Histological Type
Non-aggressive type	Endometrioid endometrial carcinoma (grades I and II) (EEC)
Aggressive types	Endometrioid endometrial carcinoma (grade III) (EEC)
	Serous carcinoma (SC)
	Undifferentiated carcinoma (UC)
	Clear cell carcinoma (CCC)
	Carcinosarcoma (CS)
	Mixed carcinoma (MC)
	Other unusual types, mesonephric-like adenocarcinoma
	Gastrointestinal mucinous type carcinoma

**Table 2:** Molecular classification of endometrial cancer according to The Cancer Genome Atlas [48].

Molecular Subgroup	Characteristics of the Subgroup	Prognosis
POLE ultra mutated subtype	- Repetitive mutation in the exonuclease domain of polymerase epsilon (POLE); - Better prognosis associated with active lymphocytic infiltration; - The recurrence of the tumor in this subgroup is lower than in other subgroups; - The amplification of ERBB2 is responsible for the POLE mutation.	Favorable prognosis
Microsatellite instability hypermutated subtype (MSI)	- Observation of mismatch repair defects with high tumor mutational burden in this category of malignancies; - Assessment of mutations in PTEN, ARID1A, PIK3CA, PIK3R1 and RPL22; - Mutation of MLH1, MSH2, MSH6, PMS2, and EPCAM, which are responsible for the microsatellite instability;	Intermediate prognosis
Copy-number low subtype	- Low copy number alteration; - Decreased mutational burden; - Mutation of CTNNB1 leading to a nuclear accumulation of β-catenin, which is associated with the Wnt pathway; - Amplification of chromosome arm 1q is related to a worse outcome.	Intermediate prognosis
Copy-number high subtype	- High number of copy number alterations; - Includes high-grade and aggressive tumors; - Frequent TP53 mutations and PIK3CA, FBXW7 and PPP2R1A, which are exclusive to this subtype.	Worst prognosis

**Table 3:** Classification of endometrial cancer according to ProMisE [50].

ProMisE Subgroup	Concordant TCGA Subgroup	Characteristics of the ProMisE Subgroup	References
MMR deficient subgroup (MMRd)	TCGA «MSI» subgroup	- Defined by the absence of one of MMR proteins (MLH1, MSH2, MSH6, PMS2, EPCAM); - Silencing mutations causing genome instability and increased microsatellite instability (MSI); - Subgroup representing 15 to 30% of recurrent/metastatic EC; - Subgroup with intermediate prognosis.	[51,52,53]
POLE mut subgroup	TCGA «POLE ultramutated» subgroup	- High immunogenicity due to the hypermutation rate and the response to therapy; - Favorable survival outcome; - Low rate of recurrence independent of adjuvant treatment;	[54,55]
p53 abn subgroup	TCGA «copy-number high» subgroup	- Most aggressive subgroup and has the worst prognosis; - Resistant to the standard therapy (surgery + chemotherapy); - High rate of somatic copy-number alterations; - Low rate of mutations; - Mutation in TP53 gene;	[50,56,57]
No specific molecular profile subgroup (NSMP)	TCGA «copy-number low» subgroup	- No mutation in TP53; - Low mutational burden; - Alteration of the PI3K/AKT and Wnt/β-catenin pathways.	[58,59]

**Table 4:** Classification of endometrial cancer according to the International Federation of Gynecology and Obstetrics (2023 update) [60].

Aggressive Cancer	Endometrioid Endometrial Carcinomas (EECs)	Low-Grade Endometrioid Carcinoma (Grade I and II)	Characterized by Excessive Estrogen Exposure
**Non-aggressive cancer**	Non-endometrioid endometrial carcinomas (NEECs)	High-grade endometrioid carcinoma (grade III), serous carcinoma (SC), clear cell carcinoma (CCC), mixed carcinoma (MC), undifferentiated carcinoma (UC), carcinosarcoma (CS), other rare forms, including mesonephric-like, and gastrointestinal mucinous carcinomas	Characterized by the absence of excessive estrogen exposure

**Table 5:** Differentiation between lymph node status according to the FIGO criteria [66].

Lymph Node Status	Size	Prognosis
Micrometastasis	0.2–2 mm and/or >200 cells	Unclear
Isolated tumor cells	≥0.2 mm and ≤200 cells	Unclear
Macrometastasis	>2 mm	Unfavorable

## Abbreviations

The following abbreviations are used in this manuscript:

CCL20	CC chemokine ligand 20
DNA	Desoxyribonucleic acid
EC	Endometrial cancer
E6-AP	E6-associated protein
FADD	Fas-associated protein with death domain
FIGO	International federation of gynecology and obstetrics
HPV	Human papillomavirus
HR-HPV	High-risk human papillomavirus
IFN	Interferon
IL	Interleukin
LR-HPV	Low-risk human papillomavirus
LVM	Low-volume metastasis
LVSI	Lymphovascular space invasion
MAPK	Mitogen-activated protein kinase
MDSC	Myeloid-derived suppressive cell
MHC	Major histocompatibility complex
MMRd	Mismatch repair deficiency
NCID	Notch intracellular domain
NK	Natural killer cell
NSMP	No specific molecular profile
p53 abn	p53 abnormal
PCR	Polymerase chain reaction
PGE2	Prostaglandin E2
POLE mut	POLE mutated
pRB	Retinoblastoma protein
PRR	pathogen recognition receptor
RT-PCR	Real-time polymerase chain reaction
SHBG	Sex-hormone-binding globulin
SLN	Sentinel lymph node
TAM	Tumor-associated macrophage
TCGA	The Cancer Genome Atlas
TGF	Transforming growth factor
TNF	Tumor necrosis factor
VEGF	Vascular endothelial growth factor
WHO	World Health Organization
